# Impact of Pandemics/Epidemics on Emergency Department Utilization for Mental Health and Substance Use: A Rapid Review

**DOI:** 10.3389/fpsyt.2021.615000

**Published:** 2021-02-24

**Authors:** Julie Munich, Liz Dennett, Jennifer Swainson, Andrew J. Greenshaw, Jake Hayward

**Affiliations:** ^1^Faculty of Medicine and Dentistry, University of Alberta, Edmonton, AB, Canada; ^2^Scott Health Sciences Library, Faculty of Medicine and Dentistry, University of Alberta, Edmonton, AB, Canada; ^3^Department of Psychiatry, Faculty of Medicine and Dentistry, University of Alberta, Edmonton, AB, Canada; ^4^Department of Emergency Medicine, Faculty of Medicine and Dentistry, University of Alberta, Edmonton, AB, Canada

**Keywords:** COVID-19, pandemic, emergency care, psychiatry, review

## Abstract

**Background:** A prolonged COVID-19 pandemic has the potential to trigger a global mental health crisis increasing demand for mental health emergency services. We undertook a rapid review of the impact of pandemics and epidemics on emergency department utilization for mental health (MH) and substance use (SU).

**Objective:** To rapidly synthesize available data on emergency department utilization for psychiatric concerns during COVID-19.

**Methods:** An information specialist searched Medline, Embase, Psycinfo, CINAHL, and Scopus on June 16, 2020 and updated the search on July 24, 2020. Our search identified 803 abstracts, 7 of which were included in the review. Six articles reported on the COVID-19 pandemic and one on the SARS epidemic.

**Results:** All studies reported a decrease in overall and MH related ED utilization during the early pandemic/epidemic. Two studies found an increase in SU related visits during the same period. No data were available for mid and late stage pandemics and the definitions for MH and SU related visits were inconsistent across studies.

**Conclusions:** Our results suggest that COVID-19 has resulted in an initial decrease in ED visits for MH and an increase in visits for SU. Given the relative paucity of data on the subject and inconsistent analytic methods used in existing studies, there is an urgent need for investigation of pandemic-related changes in ED case-mix to inform system-level change as the pandemic continues.

## Introduction

The medical burden of the SARS CoV-2 virus (COVID-19) is well-documented, but the potential for a corresponding global mental health crisis is largely understudied (1). Editorials and opinion pieces from mental health (MH) and addictions experts have sounded the alarm projecting widespread deterioration of the collective psyche and have noted the trauma brought forth by the pandemic (2, 3). Authors have emphasized the potential for increased rates of suicide citing established links between unemployment and suicide attempts (2). They have also underscored the relationship between loneliness and mental illness and hint that social isolation, a widespread public health policy to control COVID-19 infection risk, can destabilize existing MH or substance use (SU) disorders or cause new illness in those predisposed (4–6).

Community survey data from early stages of COVID-19 suggest negative shifts in population mental health, with increasing anxiety and substance use (7–9). As with many chronic diseases, MH and SU disorders require complex care models that are difficult to maintain during a pandemic and although telehealth solutions are being implemented rapidly, their effectiveness remains uncertain, especially for patients who require intensive inter-personal support. Many patients with MH or SU disorders additionally belong to underserved and vulnerable populations who typically have less access to the necessary technology and rely on the emergency department (ED) as a safety net. Even for those without pre-existing SU disorder, an increase in SU during the pandemic may lead to ED visits for intoxication or overdose. Deteriorating community-level MH may translate to ED visits for MH and SU and cause strain in the hospital system.

Rapid shifts in ED case-mix during COVID-19 have made it difficult to match capacity with need (10). While some areas experienced massive presentation rates of infected individuals such as New York, many EDs experienced an overall reduction in visits, especially in regions where prevalence remained (11). As the pandemic progresses and the population adapts to changing expectations with a phasing-in of relaxed physical distancing in many areas, EDs may expect an increase in visits related to MH and SU. Media reports suggest a wave of MH/SU ED visits is already beginning, yet there is a paucity of empirical data (12, 13).

This leads to the question; will COVID-19 cause an increase in Emergency Department (ED) utilization for MH and SU? This rapid review aims to summarize the current literature reporting COVID-19 effects on MH/SU-related ED utilization, and add value from inclusion of relevant review literature from past pandemics and epidemics.

## Methods

Rapid reviews are intended to capture a snapshot of the current literature that is rapidly changing and evolving (14). The aim of this review was to complete a synthesis of available literature within 2 months. Recognizing the rarity of pandemics and the evolving nature of COVID-19 and associated literature, our search also targeted studies from previous pandemics (e.g., H1N1) and epidemics (e.g., SARS). Ethics requirement was waived by the University of Alberta Research Ethics Board.

Search terms were developed through discussion within the research team, including a librarian specializing in health sciences (L.D.), and were designed to capture the following concepts: (1) population – patients with Mental Health issues (including mood disorders, psychoses, and suicidal ideation) and/or substance use disorders (e.g., stimulant^*^, opioid^*^, marijuana, cannabis, cocaine, heroin, fentanyl); (2) exposure – pandemics (H1N1, COVID-19) and epidemics (including COVID-19, SARS, MERS, H1N1, Ebola, and Swine Flu); (3) Setting – ED setting (e.g., emergency service, emergency medicine, trauma center, and further expanded terms).

Librarian L.D. conducted searches in Medline, Embase, Psycinfo, CINAHL, and Scopus on June 16, 2020 and updated them on July 24, 2020. We identified observational studies quantifying the association between pandemics and ED utilization for MH/SU related complaints. The search was not limited by date, language or study design; conference abstracts and theses were included where available. Bibliographies of relevant manuscripts were reviewed manually and the authors consulted with local knowledge experts to identify additional key evidence. The complete search is available in the Supplementary Table 2.

### Inclusion Criteria

The following broad inclusion criteria were applied: empirical quantitative data, exposure to an epidemic or pandemic, emergency or urgent care settings, relevant outcomes (ED visits, hospitalizations, ED case-mix). We excluded manuscripts reporting anecdotal evidence through editorials or opinions pieces. We also excluded studies in pediatric populations.

### Study Selection

References were downloaded and deduped in Endnote X7.8 (Thomson Reuters, 2016) and managed online using Covidence (Veritas Health Innovation, 2020). Abstracts were initially screened for relevance by title and then by abstract (J.H. and J.M.). Conflicts were resolved by consensus through discussion, first between the reviewers and then the larger research team if additional input was required. J.M. performed the full text screening and both J.H and J.M. reviewed the final sample of full texts. The review was not registered in PROSPERO.

### Data Extraction

Studies were extracted into Excel summary tables by reviewer J.M. and verified by J.H. Data fields were pre-defined based on discussion amongst the authors and included information about study goals, setting, design, outcomes and limitations. Regular project meetings were conducted and any differences in data extraction between the reviewers were resolved through discussion for consensus.

### Data Analysis

A thematic mapping analysis was performed to characterize the content of the final studies without attempting quantitative effect estimation. The qualitative thematic analysis involved multiple round-table discussions amongst the researchers to identify and code emerging themes, similar to a qualitative thematic analysis. Themes were refined and discussed until consensus was reached.

## Results

**Figure 1:**
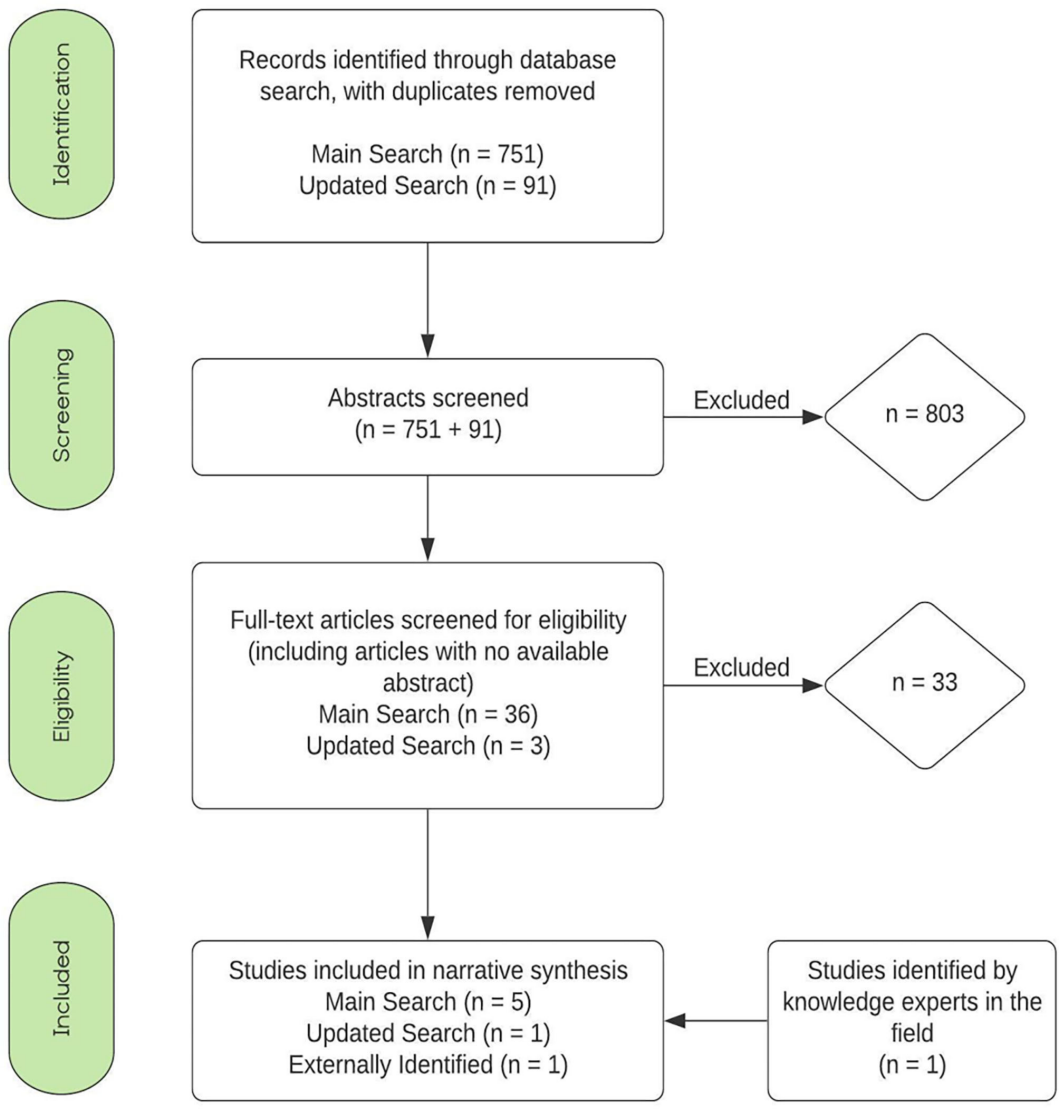
Protocol flow diagram.

Search results are reported in Figure 1; abbreviated study summaries with citations are in Table 1. The search resulted in a total of 1,308 abstracts. After removal of duplicates, 841 studies were screened for relevance based on the title and later abstract. Of these, 39 studies were included in the full text screen. The most frequent reason for exclusion was “Opinion Piece,” meaning the publication did not include any data but instead was an opinion of an expert in the field. Ultimately 6 studies were included in the final data extraction. A complete summary of study characteristics and extracted data is provided in Supplementary Table 1.

**Table 1:** Summary of study outcomes.

	**Country**	**Design**	**Exposure**	**Participants**	**Quarantine period**	**Measures**	**Findings**
Castro (15)	USA	Cross-sectional	COVID-19	205,957 visits and 2,483,159 clinical notes	January 2–March 25, 2020	Computer algorithm search for key terms to identify psychiatric complaints	Decreased frequency of psychiatric key terms in clinical notes
Goldenberg (16)	USA	Cross-sectional	COVID-19	180,893 visits	January 1–May 4, 2020	Unclear, psychiatric ED visits as percentage of all ED visits	Decreased overall and MH related visits; no decrease in psychiatric admissions.
Hartnett (17)	USA	Cross-sectional	COVID-19	1–2.5 million visits per week (~3,552 EDs)	January 1–May 30, 2020	Weekly ED visits classified by billing code.	Significant decrease in overall visits. Increased visits for 'stimulant related disorders' and 'mental health and substance use disorders in remission'
Hoyer (18)	Germany	Cross-sectional	COVID-19	Unclear, ~2250 presentations	January 1–April 19, 2020	Number of presentations to a psychiatric hospital	Decreased MH related ED visits.
Huang (19)	Taiwan	Cross-sectional	SARS CoV-1	17,586 patients	March 14–August 31, 2003	Number of ED visits, stratified by urgency	Decreased overall and “Psychiatric” visits. No change for “suicide attempt with drug overdose”
Pham-Scottez (20)	France	Cross-sectional	COVID-19	Unclear, ~4,700 presentations	March 17–May 10, 2020	Number of psychiatric consultations	Decrease in psychiatry consults.
Smalley (21)	USA	Cross-sectional	COVID-19	87,840 visits	March 25–April 24, 2020	Number of psychiatric ED visits by complaint using billing codes	Decrease in overall and suicide related visits; increase in alcohol related visits.

### Settings

All articles except one (Hartnett (17); CDC report) were published in peer-reviewed academic journals. All were retrospective studies using administrative data. Six reported on the early COVID-19 pandemic while Huang (19) reported on the SARS epidemic in 2005. Huang (19) (Taiwan), Hoyer (18) (Switzerland), Pham-Scottez (20) (France), and Goldenberg (16) (U.S.) conducted single-site studies in large urban hospital EDs while Smalley (21) (U.S.) analyzed a large multi-site sample spanning 20 EDs and >750,000 visits. Castro (15) (U.S.) also examined a large multi-site sample including over 2.5 million visits to EDs and outpatient clinics. Hartnett (17) (U.S.) studied the largest sample reporting data from over 3,000 hospitals across 47 states.

### Exposure

COVID studies analyzed the early period of the pandemic, the longest being that of Hartnett (18) which included data up to May 30 2020. All studies used a comparator time period from the preceding year to control for seasonal effects. Huang (19) study spanned the entire SARS epidemic (Mar–Aug 2003) including the pre and post-epidemic periods.

### Outcome Measures

With the exception of Castro (15), outcome metrics were similar across studies, typically measuring the total number of MH or SU related ED visits during the specified time periods and comparing this sum to the corresponding period in the preceding year. Diagnostic categories/codes underlying those counts were, however, inconsistent and incompletely described. Castro (15) tracked the number of expert- curated psychiatric key term “mentions” in clinical notes for 60,428 patients; it is not clear how many ED visits occurred, SU-related terms were not included, and key terms were not mapped to formal diagnostic codes. Only Huang (19), Smalley (21), and Hartnett (17) reported on MH/SU related visits as a *proportion* of total visits, yet descriptions of diagnostic categories were brief. Huang (19) used the “main diagnosis” from chart review to identify “psychiatric problem/disease” and “suicide attempt with drug overdose” while Hoyer (18) used the “final diagnosis after psychiatric evaluation” to compare “mental disorders” and “affective disorders; neither author elaborated on their method. Smalley (21) and Hartnett (17) both used ICD-10 codes with Smalley (21) tracking “behavioral health complaints,” “suicidal ideation”, and “alcohol related visits” while Hartnett (17) reported on “stimulant related disorders” and “mental health and substance use disorders in remission”. Goldenberg (16) and Pham-Scottez (20) did not categorize visits by diagnosis but rather tracked psychiatric visits and consultations, respectively.

### Quality

Except for Huang (19) (SARS), all publications were research letters or short reports published online in pre-print for rapid dissemination. The risk of bias was determined to be low given the use of large administrative datasets and broad inclusion criteria. Statistical analyses were generally brief, with unadjusted crude rate comparisons between pandemic periods and the corresponding year. Descriptions of analytic techniques were also brief. Definitions for MH and SU categories were generally inadequate for comparison across studies; no study listed diagnostic codes.

### Narrative Synthesis

Results from the seven studies are shown in Table 1. Across all studies, MH related and overall ED visits decreased during early pandemics/epidemics. Decreases were inversely proportional to infection rates, reaching a nadir at peak periods and returning gradually to pre-pandemic levels as infections waned. MH and SU related visit rates were impacted differently - while MH visits decreased, SU visits increased, most clearly reflected in the increased alcohol and stimulant related visits reported by Smalley (21) and Hartnett (17), respectively. In the remaining studies, MH and SU diagnoses were often combined in a diagnostic grouping making inferences difficult. For instance, Huang (19) found that “suicide attempt with drug overdose” increased while “psychiatric problem/disease” decreased. In some of these cases the SU related effect may have predominated leading to increased visits for the category overall. Thus, to the extent examined, SU related visits seem to increase in the context of a general decrease in ED demand.

## Discussion

This review shows that MH related ED visits decrease during early pandemics/epidemics, in line with an overall decrease in ED demand. In contrast, SU related visits appear to increase. In most cases, however, studies fail to reliably differentiate MH from SU producing inconsistent and difficult to interpret results. MH diagnoses are rarely sub-stratified and are often combined with SU diagnoses. Reports on ED utilization from large US hospital systems, which do not analyze MH and SU specifically, confirm a dramatic decrease in ED demand across the US throughout the early pandemic periods (22). Reduced ED visits, including those that are MH related, may reflect a broad-based avoidance of hospitals and/or the emergence of telehealth remote care solutions. However, explanations remain conjecture given the dearth of high-quality studies.

Our results highlight the need for continued work in this domain. The international research community must undertake efforts to standardize case definitions for mental and physical disorders based on administrative data to allow for comparison of results as we enter the mid and late stage pandemic. Later stage observations from SARS in China (16) are of limited generalizability to COVID-19 given the unprecedented extent of current global disruption. Although the H1N1 pandemic was included in our search timeframe, no relevant data emerged, likely because H1N1 also had a limited impact on society overall. Thus, we find ourselves in uncharted territory where modern analytic techniques have never previously been employed. A prolonged pandemic characterized by intermittent outbreaks, economic recession, frayed social networks and changing public health measures is clearly the substrate for increased mental health suffering and demand for ED services.

### Limitations

Our search did not include gray literature or non-English publications. The study protocol was designed for rapid synthesis and dissemination of results and did not allow for quantitative analysis. Given time constraints, we were unable to complete a systemic evaluation of study quality. We believe these limitations do not undermine the study's impact of our review given the limited data available for review.

## Conclusions

There is a desperate need for study informing a system-level response to COVID-19 that matches capacity with need. No high-quality data exist to guide preparation for the mid and late pandemic stages. While the pace of publication during early COVID-19 is impressive, information is arriving too slowly to inform system-level change. Future work must focus on clarifying the complex effects of pandemics/epidemics on MH and SU related healthcare utilization and, where possible, building predictive models for condition-specific ED demand so that resources can be mobilized when and where they are most needed.

## Data Availability Statement

The original contributions presented in the study are included in the article/Supplementary Material, further inquiries can be directed to the corresponding author.

## Author Contributions

JM, JS, AG, and JH conceived of the study idea. LD, AG, JM, and JH contributed to study design, data collection, and analysis. LD carried out the literature search. JH and JM wrote the manuscript. JH supervised the project. All authors have critically reviewed the manuscript.

## Conflict of Interest

The authors declare that the research was conducted in the absence of any commercial or financial relationships that could be construed as a potential conflict of interest.
