# Supplementary material for: Impact of Pandemics/Epidemics on Emergency Department Utilization for Mental Health and Substance Use: A Rapid Review
Source: Front Psychiatry. 2021 Feb 24;12:615000. doi: 10.3389/fpsyt.2021.615000 (PMC7943839; doi:10.3389/fpsyt.2021.615000)
Supplement: Supplementary file 1 [file Table_1.DOCX]

**Supplementary Table S1: Search Strategy**

**Ovid MEDLINE(R) ALL 1946 to July 23, 2020**

Date searched: June 16, 2020 Updated July 24, 2020

Results: 246

Search saved as: Mental health in the ER during pandemic - Medline

1. ((disease outbreaks/ or epidemics/) not (silent epidemic* or ((vitamin D or burnout or opioid* or opiate* or narcotic* or fentanyl or overdose* or substance* or illicit drug* or injection drug* or heroin or addiction*) adj20 epidemic*)).mp.) or pandemics/

2. Severe Acute Respiratory Syndrome/

3. coronaviridae infections/ or coronavirus infections/

4. coronaviridae/ or coronavirus/ or betacoronavirus/ or coronavirus oc43, human/ or middle east respiratory syndrome coronavirus/ or sars virus/

5. influenza a virus, h1n1 subtype/

6. Ebolavirus/

7. Hemorrhagic Fever, Ebola/

8. (Coronavirus* or corona-virus* or ncoV* or n-cov* or 2019-ncov or covid or covid19 or covid2019 or SARS-CoV* or SARSCov* or pandemic* or Outbreak* or SARS or Severe Acute Respiratory Syndrome or MERS or MERSCov* or middle east respiratory syndrome or H1N1 or swine flu or swine influenza or Ebola).mp.

9. ((Epidemic* or public health emergenc*) not (silent epidemic* or ((vitamin D or burnout or opioid* or opiate* or narcotic* or fentanyl or overdose* or substance* or illicit drug* or injection drug* or heroin or addiction*) adj20 (epidemic* or public health emergenc*)))).mp.

10. (social* distan* adj10 (effect or effects or policy or policies or requirement*)).mp.

11. quarantine/ or (quarantine* or lockdown*).mp.

12. or/1-11

13. limit 12 to editorial

14. 12 not 13

15. Emergency Treatment/ or Emergency Medicine/ or emergency medical services/ or emergency service, hospital/ or trauma centers/ or triage/ or exp Evidence-Based Emergency Medicine/ or exp Emergency Nursing/ or Emergencies/ or (ER or ED or emergicent* or casualty department* or ((emergenc* or casualty) adj1 (room* or accident or ward or wards or unit or units or department* or physician* or doctor* or nurs* or treatment* or patient*)) or (trauma adj1 (cent* or care)) or acute care or urgent care).mp.

16. Mental Health/

17. exp Mental disorders/

18. (mental health or mental* ill* or depression or depressive or anxiety disorder* or psychoses or psychosis or psychotic* or suicid* or compulsive disorder* or OCD or bipolar or mania or adjustment disorder* or traumatic stress or panic disorder* or mood disorder* or situational cris*).mp.

19. (((stimulant* or substance* or opioid* or marijuana or cannabis or cocaine or heroin or illicit-drug* or fentanyl) adj3 ("use" or user* or usage or abuse* or misuse* or addict*)) or drug abuse* or addiction* or addictive behavio*).mp.

20. psychiatric.mp.

21. dual diagnos*.mp.

22. or/16-21

23. emergency services, psychiatric/

24. 14 and ((15 and 22) or 23)

**Embase 1974 to 2020 July 23 (OVID interface)**

Date searched: July 24, 2020

Results: 589

Search saved as: Mental health in the ER during pandemic - Embase

1. epidemic/ not (silent epidemic* or ((vitamin D or burnout or opioid* or opiate* or narcotic* or fentanyl or overdose* or substance* or illicit drug* or injection drug* or heroin or addiction*) adj20 epidemic*)).mp.

2. pandemic/

3. pandemic influenza/ or swine influenza/

4. coronavirus infection/ or middle east respiratory syndrome/ or severe acute respiratory syndrome/

5. exp coronavirinae/

6. exp ebolavirus/

7. Ebola hemorrhagic fever/

8. (Coronavirus* or corona-virus* or ncoV* or n-cov* or 2019-ncov or covid or covid19 or covid2019 or SARS-CoV* or SARSCov* or pandemic* or Outbreak* or SARS or Severe Acute Respiratory Syndrome or MERS or MERSCov* or middle east respiratory syndrome or H1N1 or swine flu or swine influenza or Ebola).mp.

9. ((Epidemic* or public health emergenc*) not (silent epidemic* or ((vitamin D or burnout or opioid* or opiate* or narcotic* or fentanyl or overdose* or substance* or illicit drug* or injection drug* or heroin or addiction*) adj20 (epidemic* or public health emergenc*)))).mp.

10. (social* distan* adj10 (effect or effects or policy or policies or requirement*)).mp.

11. quarantine/

12. (quarantine* or lockdown*).mp.

13. or/1-12

14. limit 13 to editorial

15. 13 not 14

16. emergency health service/ or hospital emergency service/

17. emergency treatment/ or emergency care/ or evidence based emergency medicine/

18. emergency medicine/ or evidence based emergency medicine/ or emergency nursing/ or emergency ward/

19. ((ER or ED).tw. and emergency.mp,jx.) or (emergicent* or casualty department* or ((emergenc* or casualty) adj1 (room* or accident or ward or wards or unit or units or department* or physician* or doctor* or nurs* or treatment* or patient*)) or (trauma adj1 (cent* or care)) or acute care or urgent care).mp.

20. or/16-19

21. mental health/ or psychological well-being/

22. exp mental disease/

23. (mental health or mental* ill* or depression or depressive or anxiety disorder* or psychoses or psychosis or psychotic* or suicid* or compulsive disorder* or OCD or bipolar or mania or adjustment disorder* or traumatic stress or panic disorder* or mood disorder* or situational cris*).mp.

24. (((stimulant* or substance* or opioid* or marijuana or cannabis or cocaine or heroin or illicit-drug* or fentanyl) adj3 ("use" or user* or usage or abuse* or misuse* or addict*)) or drug abuse* or addiction* or addictive behavio*).mp.

25. (psychiatric or dual-diagnos*).mp.

26. or/21-25

27. psychiatric emergency service/

28. 15 and ((20 and 26) or 27)

**APA PsycInfo 1806 to July Week 3 2020 (OVID interface)**

Date searched: July 24, 2020

Results:54

Search saved as: Mental health in the ER during pandemic - Psycinfo

1. epidemics/ not (silent epidemic* or ((vitamin D or burnout or opioid* or opiate* or narcotic* or fentanyl or overdose* or substance* or illicit drug* or injection drug* or heroin or addiction*) adj20 epidemic*)).mp.

2. pandemics/

3. swine influenza/

4. (Coronavirus* or corona-virus* or ncoV* or n-cov* or 2019-ncov or covid or covid19 or covid2019 or SARS-CoV* or SARSCov* or pandemic* or Outbreak* or SARS or Severe Acute Respiratory Syndrome or MERS or MERSCov* or middle east respiratory syndrome or H1N1 or swine flu or swine influenza or Ebola or quarantine* or lockdown*).mp.

5. ((Epidemic* or public health emergenc*) not (silent epidemic* or ((vitamin D or burnout or opioid* or opiate* or narcotic* or fentanyl or overdose* or substance* or illicit drug* or injection drug* or heroin or addiction*) adj20 (epidemic* or public health emergenc*)))).mp.

6. (social* distan* adj10 (effect or effects or policy or policies or requirement*)).mp.

7. or/1-6

8. limit 7 to ("column/opinion" or editorial or review-book or review-media or review-software & other)

9. 7 not 8

10. emergency services/ or emergency medicine/

11. (ER or (ED not (eating disorder* or erectile dysfunction)) or emergicent* or casualty department* or ((emergenc* or casualty) adj1 (room* or accident or ward or wards or unit or units or department* or physician* or doctor* or nurs* or treatment* or patient*)) or (trauma adj1 (cent* or care)) or acute care or urgent care).tw.

12. 10 or 11

3. exp mental health/

14. exp mental disorders/

15. (mental health or mental* ill* or depression or depressive or anxiety disorder* or psychoses or psychosis or psychotic* or suicid* or compulsive disorder* or OCD or bipolar or mania or adjustment disorder* or traumatic stress or panic disorder* or mood disorder* or situational cris* or psychiatric or dual-diagnos*).tw.

16. (((stimulant* or substance* or opioid* or marijuana or cannabis or cocaine or heroin or illicit-drug* or fentanyl) adj3 ("use" or user* or usage or abuse* or misuse* or addict*)) or drug abuse* or addiction* or addictive behavio*).mp.

17. or/13-16

18. 9 and 12 and 17

**CINAHL Plus with Full text (EBSCOhost interface)**

Date searched: July 24, 2020

Results:160

Deselect: Apply equivalent subjects

Search saved as: Mental health in the ER ONLY during pandemic - CINAHL in ldennett account

S1 (((MH "Disease Outbreaks") or epidemic* or public-health-emergenc*) NOT (silent-epidemic* or ((vitamin-D or burnout or opioid* or opiate* or narcotic* or fentanyl or overdose* or substance* or illicit drug* or injection drug* or heroin or addiction*) N20 (epidemic* or public-health-emergenc*))))

S2 (MH "Influenza, Pandemic (H1N1) 2009") OR (MH "Influenza, Swine")

S3 Coronavirus* or corona-virus* or ncoV* or n-cov* or 2019-ncov or covid or covid19 or covid2019 or SARS-CoV* or SARSCov* or pandemic* or Outbreak* or SARS or Severe-Acute-Respiratory-Syndrome or MERS or MERSCov* or middle-east-respiratory-syndrome or H1N1 or swine-flu or swine-influenza or Ebola or quarantine* or lockdown*

S4 S1 OR S2 OR S3

S5 (MH "Psychiatric Emergencies")

S6 (MH "Physicians, Emergency") OR (MH "Emergency Service+") OR (MH "Emergency Medicine") OR (MH "Emergency Nurse Practitioners")

S7 (emergicent* or casualty-department* or ((emergenc* or casualty) N1 (room* or accident or ward or wards or unit or units or department* or physician* or doctor* or nurs* or treatment* or patient*)) or (trauma N1 (cent* or care)) or acute-care or urgent-care)

S8 S6 OR S7

S9 ( (MH "Mental Disorders+") OR (MH "Mental Health Services") OR (MH "Mental Health") ) OR ( mental-health or mental*-ill* or depression or depressive or anxiety-disorder* or psychoses or psychosis or psychotic* or suicid* or compulsive-disorder* or OCD or bipolar or mania or adjustment-disorder* or traumatic-stress or panic-disorder* or mood-disorder* or situational-cris* or psychiatric or dual-diagnos* ) OR ( (((stimulant* or substance* or opioid* or marijuana or cannabis or cocaine or heroin or illicit-drug* or fentanyl) N3 ("use" or user* or usage or abuse* or misuse* or addict*)) or drug-abuse* or addiction* or addictive-behavio*) )

S14 S4 AND (S5 OR (S8 AND S9))

**SCOPUS**

Date searched: July 24, 2020

Results: 259

Search#1 for ERs

TITLE-ABS-KEY ( ( epidemic* OR public-health-emergenc* ) AND NOT ( silent-epidemic* OR ( ( vitamin-d OR burnout OR opioid* OR opiate* OR narcotic* OR fentanyl OR overdose* OR substance* OR illicit-drug* OR injection-drug* OR heroin OR addiction* ) W/20 ( epidemic* OR public-health-emergenc* ) ) ) ) OR TITLE-ABS-KEY ( coronavirus* OR corona-virus* OR ncov* OR n-cov* OR 2019-ncov OR covid OR covid19 OR covid2019 OR sars-cov* OR sarscov* OR pandemic* OR outbreak* OR sars OR severe-acute-respiratory-syndrome OR mers OR merscov* OR middle-east-respiratory-syndrome OR h1n1 OR swine-flu OR swine-influenza OR ebola OR quarantine* OR lockdown* ) AND TITLE-ABS-KEY ( emergicent* OR casualty-department* OR ( ( emergenc* OR casualty ) W/1 ( room* OR accident OR ward OR wards OR unit OR units OR department* OR physician* OR doctor* OR nurs* OR treatment* OR patient* ) ) OR ( trauma W/1 ( cent* OR care ) ) OR acute-care OR urgent-care ) AND TITLE-ABS-KEY ( mental-health OR mental*-ill* OR depression OR depressive OR anxiety-disorder* OR psychoses OR psychosis OR psychotic* OR suicid* OR compulsive-disorder* OR ocd OR bipolar OR mania OR adjustment-disorder* OR traumatic-stress OR panic-disorder* OR mood-disorder* OR situational-cris* OR psychiatric OR dual-diagnos* OR ( ( stimulant* OR substance* OR opioid* OR marijuana OR cannabis OR cocaine OR heroin OR illicit-drug* OR fentanyl ) W/3 ( "use" OR user* OR usage OR abuse* OR misuse* OR addict* ) ) OR drug-abuse* OR addiction* OR addictive-behavio* )
