# Supplementary material for: Impact of Pandemics/Epidemics on Emergency Department Utilization for Mental Health and Substance Use: A Rapid Review
Source: Front Psychiatry. 2021 Feb 24;12:615000. doi: 10.3389/fpsyt.2021.615000 (PMC7943839; doi:10.3389/fpsyt.2021.615000)
Supplement: Supplementary file 2 [file Table_2.DOCX]

**Supplementary Table S2: Study Details**

| **Authors (Year)** | **Title** | **Journal** | **Location** | **Study Period** | **Pandemic Stage / Social Isolation Intensity** | **Population** | **Purpose/Objectives** | **Study Design/Methods** | **Exposure** | **Outcome** | **Main Findings** |
| --- | --- | --- | --- | --- | --- | --- | --- | --- | --- | --- | --- |
| Castro, V. M. & Perlis, R. H. (2020) | Electronic Health Record Documentation of Psychiatric Assessments  in Massachusetts Emergency Department and Outpatient Settings During the Coronavirus Disease 2019 (COVID-19) Pandemic | JAMA Network, Open | Boston, Massachusetts. USA | January 2 - March 25, 2020 | Acute phase of the COVID-19 pandemic, not defined. | All comers to ED. | To quantify shifts in psychiatric evaluation associated with COVID-19 to inform the need for telemedicine solutions. | Retrospective cohort. Searched clinical notes for key terms related to mood disorders, anxiety disorders, suicidal ideation, psychosis. Logistic regression analysis to examine association between psychiatric complaints and COVID testing. | COVID-19 | Proportion of ED notes mentioning psychiatric concerns during COVID-19 compared to years prior. | Frequency of psychiatric key terms decreased during COVID-19. Patients with psychiatric complaints were less likely to be tested for COVID. |
| Goldenberg, M. N. & Parwani, V. (2020) | Psychiatric emergency department volume during Covid-19 pandemic | The American Journal of Emergency Medicine | New Haven, Connecticut. USA | January 1 - May 4, 2020 | Not defined. | Adults ED patients to three specific hospitals | To determine if there was a change in numbers of psychiatric visits and admissions to ED during COVID-19. | Retrospective cohort. Letter to the editor. Data on Ed utilization compared across three years. | COVID-19 | Proportion of ED visits with psychiatric complaints and total ED visits during COVID-19 compared to prior years. | Decrease in ED visits overall and psychiatric ED visits; no decrease in psychiatric admissions suggesting an increased average psychiatric acuity. |
| Hartnett, K., *et al.*(2020) | Impact of the COVID-19 Pandemic on Emergency Department Visits - United States, January 1, 2019 - May 30, 2020 | CDC Morbidity and Mortality Weekly Report (MMWR) | USA | January 1 - May 30, 2020 | Early pandemic | All comers to ED. | To quantify the impact of COVID-19 on the number of ED visits in the US overall and for specific diseases. | Retrospective cohort. Analysis of National Syndromic Surveillance data with comparison to previous year. | COVID-19 | Number of overall and disease-specific ED visits per week | 42% decrease in ED visits overall. Increase in visits for 'stimulant related disorders' and 'mental health and substance use disorders in remission' |
| Hoyer, C., *et al.*(2020) | Decreased utilization of mental health emergency service during the COVID-19 pandemic | European Archives of Psychiatry and Clinical Neuroscience | Mannheim, Germany | January 1 - April 19, 2020 | WHO declared pandemic and when partial lockdown began (week 12) as two markers. | All comers to ED. | To quantify the effect of COVID-19 on emergency service utilization for mental health and to assess impact of partial lockdown in Germany. | Retrospective cohort. Short communication. Primary analysis compared ED utilization rates for psychiatric complaints across time periods (Poisson regression, Spearman’s rho). Secondary analysis of distance travelled to hospital to estimate effect of social distancing. | COVID-19 | Number of ED visits for mental health compared to prior years. | Decrease in mental health related visits during COVID-19 compared to the year prior. Greatest impact in the later phases when movement was shown to be restricted to the greatest degree by social distancing. |
| Huang, C., *et al.*(2005) | Impact of severe acute respiratory syndrome (SARS) outbreaks on the use of emergency department medical resources | Journal of the Chinese Medical Association | Northern Taiwan | Pre-epidemic (before March 14, 2003), early epidemic (March 14 - April 21, 2003), peak epidemic (April 22 - May 19), late epidemic (May 20 - June 17, 2003), and post-epidemic (June 18 - August 31, 2003). | | All comers to ED age > 14y. | To evaluate the impact of the SARS epidemic on ED utilization. | Retrospective cohort. Chart review. Main diagnosis on ED chart used to categorize visit types. | SARS CoV-1 | Number of ED visit (overall and disease-specific) during various stages of SARS and compared to pre-SARS period. | 14.7% decrease in overall ED visits in 2003 (SARS) compared to 2002 (pre-SARS). Trend toward increased visits for ‘attempted suicide by drug overdose’ |
| Pham-Scottez, A., *et al.* (2020) | Patient flow in the largest French psychiatric emergency centre in the context of the COVID-19 pandemic | Psychiatry Research | Paris, France | March 17 - May 10, 2020. | Confinement declared. | All comers to psychiatric ED. | To quantify the impact of COVID-19 and resulting isolation orders on the number of psychiatric consultations at a psychiatric emergency centre. | Retrospective cohort. Comparison of mean number of consultations per day during pre vs. post confinement periods (T tests). | COVID-19 | Number of psychiatric consults during COVID-19 compared with the same time period in previous three years, and the period immediately prior to COVID-19.. | 60% decrease in psychiatric ED visits during confinement period compared to earlier time periods. |
| Smalley, C., *et al.* (2020) | The impact of COVID-19 on suicidal ideation and alcohol presentations to emergency departments in a large healthcare system | The American Journal of Emergency Medicine | USA | March 25 - April 24, 2020. | Stay at home orders issued. | All comers to ED. | Assess the impact of social distancing/stay at home orders on prevalence of mental health disorders in the ED. | Retrospective multicentre cohort. Letter to the editor. Used billing codes to determine reason for visit. Looked for behavioural complaints, suicidal ideation, alcohol-related.. | COVID-19 | ED visits for suicidal ideation, alcohol, behavioural complaints compared to the same time period in prior years. | Decreased visits for suicidal ideation. Increased alcohol related visits. Decreased ED visits overall. |
